# Wettability, water absorption and water storage in rosette leaves of the dragon tree (*Dracaena draco* L.)

**DOI:** 10.1007/s00425-020-03433-y

**Published:** 2020-07-28

**Authors:** Joanna Jura-Morawiec, Jan Marcinkiewicz

**Affiliations:** Polish Academy of Sciences Botanical Garden-Centre for Biological Diversity Conservation in Powsin, Prawdziwka 2, 02-973 Warsaw, Poland

**Keywords:** Anatomy, Arborescent monocots, Contact angle, Hydrenchyma, Leaf water uptake

## Abstract

**Main conclusion:**

Leaf surfaces of *Dracaena draco* are wettable and can absorb water. The thick, basal leaf part could act as a water reservoir that changes in volume with plant hydration.

**Abstract:**

Rosettes of leaves of *Dracaena draco* play an important role in directing fog water through leaf axils into the stem tissues, where it can be stored for further use. However, how water is intercepted and collected by the leaves remains unclear, especially since leaf blade surfaces are considered hydrophobic. Based on the observations of *D. draco* individuals growing in Spain and in glasshouse conditions in Poland, we hypothesised that their long leaves (~ 70 cm) are able to absorb water along the whole leaf blade, but leaf age affects this process. We used water droplet contact angle measurements, anatomical analyses of leaf cross sections along the age gradient and dye tracer experiments to test this hypothesis. The data showed that the leaf surfaces of *D. draco* are wettable. In general, the mature leaves of the rosette are more wettable than the young ones. Water can be absorbed both through the adaxial and abaxial surfaces. The hydrenchyma is not uniformly distributed along the leaf, it is especially abundant towards the leaf base where it forms a massive water reservoir, which changes in volume depending on plant water status. The results of these studies shed light on the role of rosettes in water absorption by *D. draco*, and broaden our understanding of the functioning of this vulnerable species.

**Electronic supplementary material:**

The online version of this article (10.1007/s00425-020-03433-y) contains supplementary material, which is available to authorized users.

## Introduction

**Fig. 1:**
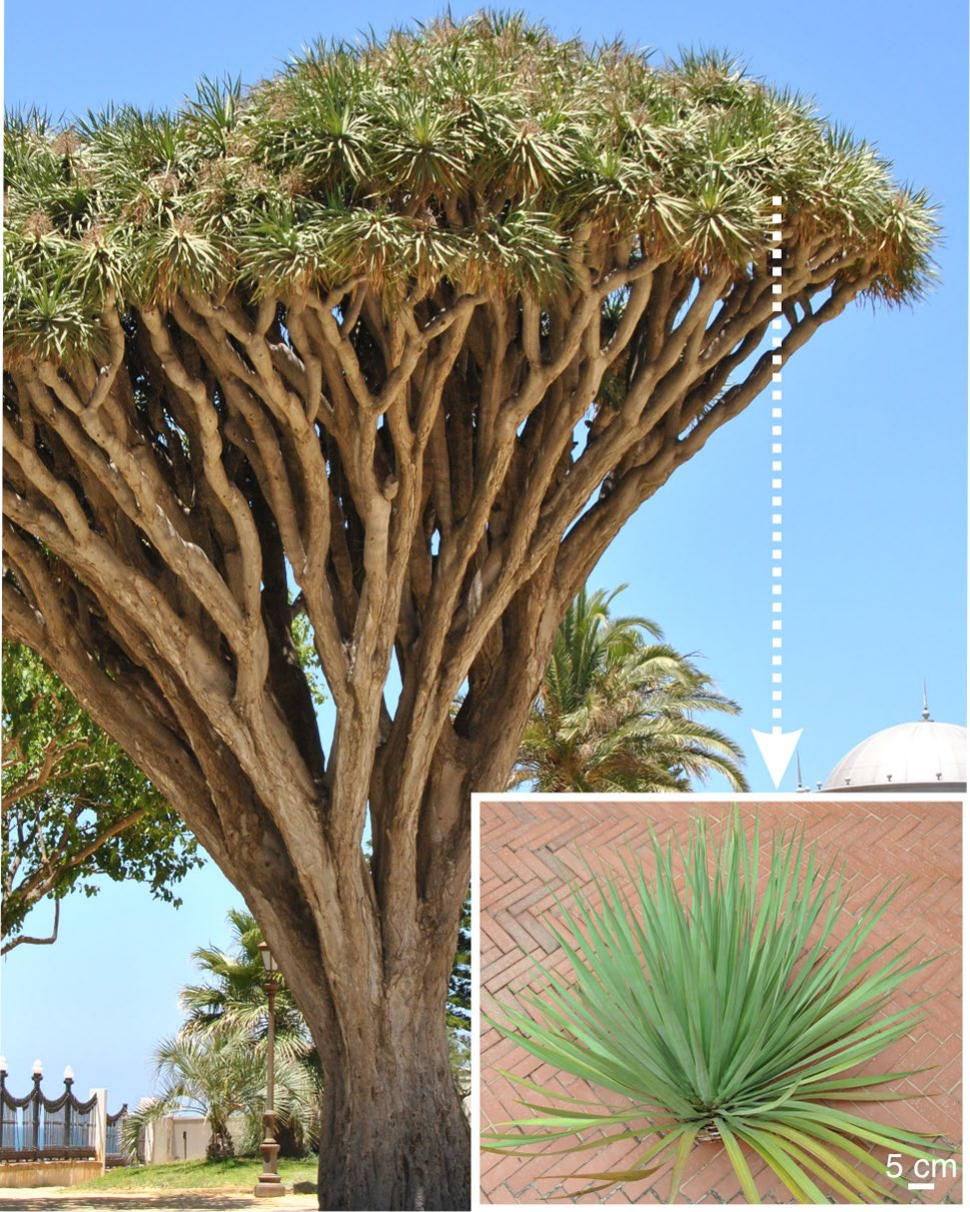
A tree-like monocot *D. draco*, in Cadiz, with rosettes of leaves located at the tips of the branches. Inset photo shows a rosette from a dragon tree growing in Jardín Botánico "Viera y Clavijo" in Gran Canaria

The dragon tree, *Dracaena draco* L., is a giant, tree-like monocotyledon which may grow to as much as 20 m in height. Its trunk branches sympodially and forms an umbrella-like canopy (Beyhl 1995), with apical rosettes of long (40–90 cm) and flexible leaves (Marrero et al. 1998; Fig. 1). *D. draco* is a vulnerable species, with distribution restricted to the Canary Islands, Cape Verde Islands, Madeira and Morocco (Marrero et al. 1998). It grows in areas that are affected by northeast trade winds, with rains in winter and relatively dry summers. Fog is a common occurrence and annual rainfall totals about 400 mm (Marrero et al. 1998; Marzol et al. 2011). On the Canary Islands, fog is possible throughout the year, but especially between June and August, i.e., during the rainless, dry season (Marzol et al. 2011). *D. draco* is adapted to cope with seasonal shortages of water. Its trunk and branches increase in girth due to monocot cambium activity (Carlquist 2012; Jura-Morawiec et al. 2015), and possess parenchyma-rich structure capable of storing water. The leaves of *D. draco* may also store water. Although the anatomy of only the mid-sections of *D. draco* leaves has been studied, we know that the leaves are slightly succulent and contain hydrenchyma, an achlorophyllous water-storage tissue (Klimko et al. 2018).

According to Kitching (2000) *Dracaena* spp. belong to plants containing water-holding leaf axils that become phytotelmata. In an experimental study by Nadezhdina and Nadezhdin (2017), in which water was applied directly to the leaf axils of young *D. draco* plants, it was shown that water absorbed through basal leaf tissues was directed to the stem, where it filled internal water reserves. Nadezhdina and Nadezhdin (2017) propose that this mechanism of water absorption, which is independent of soil water, can be especially important for the survival of dragon tree species, native to foggy but seasonally dry habitats.

Absorption of water by leaves (foliar water uptake) is a common mechanism used by plant species across the world and is of significant ecophysiological importance (Rosado and Holder 2013; Eller et al. 2016; Dawson and Goldsmith 2018; Berry et al. 2019; Schreel and Steppe 2020). Water penetration of leaf surfaces is possible through stomata (Burghardt et al. 2012), trichomes (Fernández et al. 2014), veins (Bahamonde et al. 2018), scales or through the cuticle (reviewed by Fernández et al. 2017). In general, alike leaves of many other species, *D. draco* leaves are considered incapable of absorbing water directly through the leaf blade surface. They are amphistomatous, glabrous (have no trichomes) and have a thick cuticle with epicuticular waxes deposited onto their surface (Klimko and Wiland-Szymańska 2008; Nadezhdina et al. 2015; Klimko et al. 2018). Thus they are described as rather repellent than water absorbent (Nadezhdina and Nadezhdin 2017). However, the thickness of the cuticle and the amount of wax are not related to water permeability of leaves (Schreiber and Riederer 1996). The cuticle has a heterogeneous structural and chemical nature and consists of hydrophobic compounds, waxes/cutin, as well as hydrophilic compounds, polysaccharides (Fernández et al. 2016, 2017) which determine leaf surface properties, including the possibility of water absorption, that may change with leaf age due to the influence of environmental factors (Burgess and Dawson 2004; Taylor 2011; Wang et al. 2013; Fernández et al. 2014).

The aim of our study was to analyse the morpho-anatomical properties of leaves from rosettes of *D. draco* to evaluate their ability to absorb atmospheric water. Based on our morphological observations of dragon trees growing in Spain (Gran Canaria, Cadiz, Barbate) and in glasshouse conditions in Poland, we hypothesised that long leaves of *D. draco* are able to absorb water along the blade, but leaf age affects this process. To test this hypothesis, we examined the anatomy of leaf cross sections in the basal, middle and tip regions. Next, we evaluated leaf wettability that provides insights into potential surface-related processes, such as leaf absorption of water (Brewer et al. 1991; Aryal and Neuner 2010). An experiment using fluorochromes was also performed to investigate the fate of water applied to the surface of the middle part of leaf blades. The results of these studies shed light on the role of rosettes in atmospheric water absorption by *D. draco*.

## Materials and methods

### Plant material

This study was conducted with: (i) a rosette excised from a *ca.* 40-year-old *D. draco* individual growing outdoors at the Jardin Botanico Canario "Viera y Clavijo" in Gran Canaria, and (ii) 16 leaves of three younger individuals (*ca.* 2 m, 2.5 m, 5 m tall) growing in the glasshouse of the Polish Academy of Sciences Botanical Garden (CBD, Powsin). The studies were supported with field observations of leaf rosettes morphology of dragon trees of different ages growing in Gran Canaria, in Cadiz and Barbate (Spain).

### Morpho-anatomical analysis

For excised rosette, leaves were sequentially removed and the length, width, and thickness of the basal and middle regions of each leaf were measured. Anatomical examinations were carried out on selected leaves of different ages (in case of a rosette, the leaf position is equivalent to the leaf age) using hand-cut cross sections and 40–60 µm thick sections made with a core-microtome (WSL, Birmensdorf, Switzerland). The sections were stained with safranin O (Roth) and astra blue (Roth) [v/v; 1:1], dehydrated in ethanol (50–100%) and mounted in Euparal (Roth). Stained sections were examined in transmitted light with an Olympus BX41 microscope. Pictures of cross sections were taken with a Sony A6300 camera. Unstained, hand-cut, cross sections of the middle leaf region were examined under UV light with an LED fluorescence Zeiss Axio.Lab1 microscope, equipped with a camera (Opta-Tech, Poland). Some hand-cut cross sections were also examined using the FEI Quanta 200 ESEM scanning electron microscope with the EDS EDAX analyser. Electron microscope observations and photos were taken in low vacuum mode (up to 1Tr).

### Contact angle measurements

To determine leaf wettability, young and mature leaves were sampled from the rosette (Fig. S1) and mounted horizontally on a stage using double-sided tape. A 2 µl droplet of distilled water was then placed on the leaf surface using a micropipette, and a photographic digital image of the droplet on the leaf surface was taken with a Telecentric Optical System Nikon (158.2 mm, 0.03). Based on the images, the contact angle (*θ*) between the water droplet and leaf surface (a line at a tangent to the droplet and the leaf surface) was calculated using ImageJ software. Measurements were taken on both adaxial and abaxial leaf surfaces (30 per leaf, 360 measurements in total). A wettable surface is defined as having *θ* < 90°, while a non-wettable surface is considered to have *θ* > 90°. Mean, minimum and maximum values of *θ* were calculated with Microsoft Excel. Contact angle measurements were carried out for leaves of plants growing in the glasshouse of the Polish Academy of Sciences Botanical Garden. However, water droplet behaviour on sprayed leaves was also analysed with the unaided eye on dragon trees growing outdoors in Spain.

### Leaf water absorption—dye experiments

To evaluate whether water penetrates into leaves, we performed a glasshouse experiment exposing leaves of *D. draco* to fluorescent tracer substances: fluorescein (Merck) and acridine orange (Merck). Water solutions (0.01%) of each dye were placed in the middle part of two leaves, (fluorescein on the adaxial side only; acridine orange on both leaf sides), sealed carefully with parafilm to prevent spillage of the dye, and carefully protected with aluminium foil to prevent loss of dye fluorescence properties. After 24 h and 48 h, the treated parts of leaves were excised and hand sectioned, with the resulting leaf cross sections observed using a Nikon Microphot SA fluorescence microscope (Light source HBO100W, excitation filter B-2A). The experiment was repeated twice.

## Results

### General morphology of the *D. draco* rosette

The rosette we examined was made up of 155 leaves. In general, the inclination of leaves gradually changes with leaf age. The uppermost (young) leaves are almost vertical, while the mature ones are horizontal or at an obtuse (> 90^°^) angle to the rosette tip (Figs. 1, S1). Leaf length varied from 50.7 cm to 67.8 cm. Basal leaf width increased gradually with leaf age (position within the rosette) and varied from 2.8 cm (young leaves) to 8 cm (mature leaves). The thickness of the leaf base varied from 0.6 mm to 1.2 mm, with the increase in thickness not linear among the leaves of the rosette. The middle section of the leaf blades was considerably thinner (*ca*. 3 mm) than the basal one. The basal parts of the leaves within the rosette were covered with red resin named dragon's blood.

### Rosette leaf anatomy and changes in morphology related to water content

**Fig. 2:**
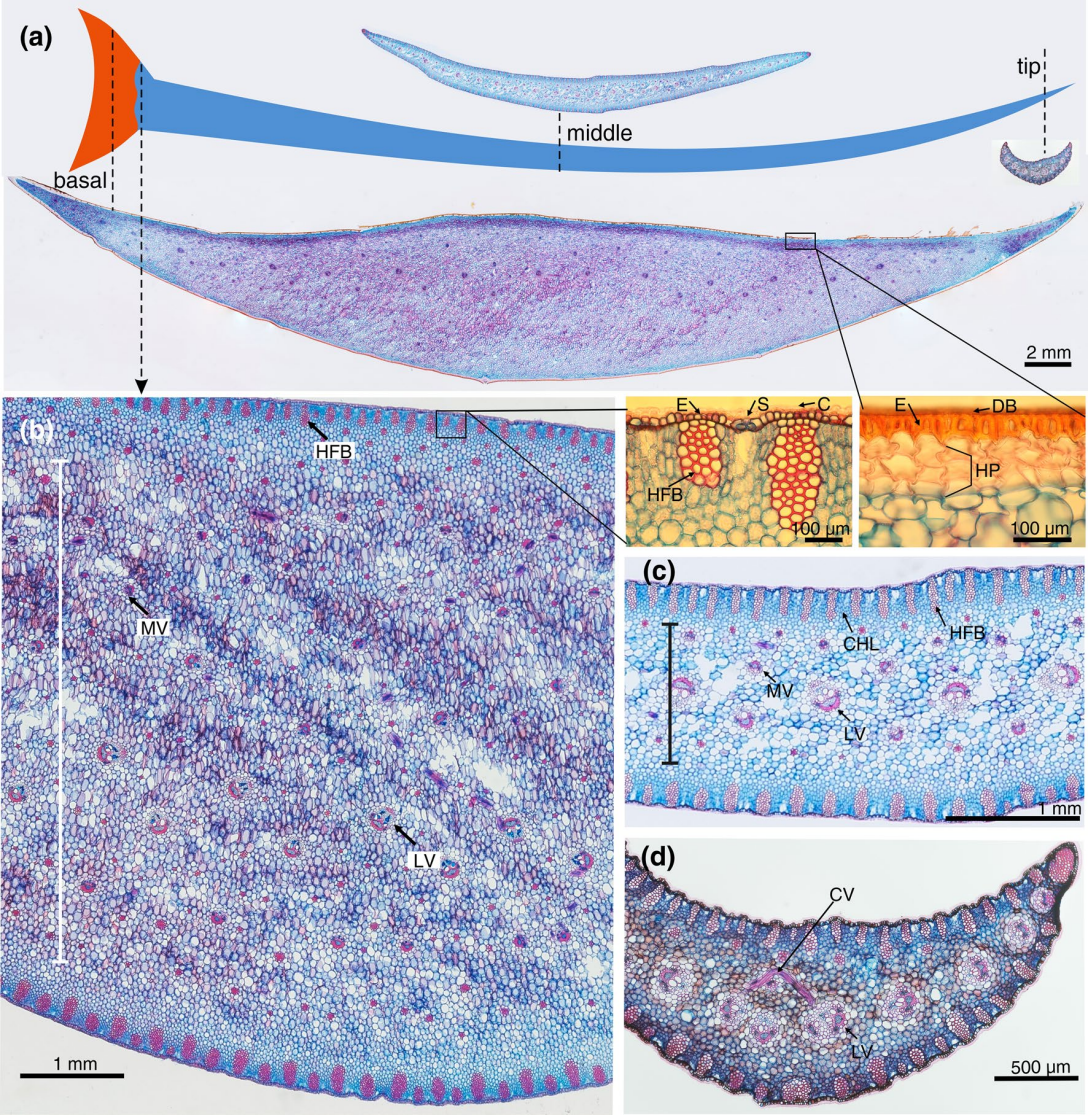
Anatomical characteristics of *D. draco* leaves. **a** Transverse sections from the basal, middle and tip of leaf shown at the same scale. **b–d** Enlarged sections of leaf basal (**b**), middle (**c**) and tip (**d**) cross sections. Note the range of mesophyll able to store water, marked with a continuous, vertical line in (**b**, **c**). Boxes indicate enlarged sections showing the differences in the superficial tissues of the basal parts of leaves uncovered and covered with dragon's blood. *C* cuticle, *CV* cross vein interconnecting longitudinal veins, *DB* dragon's blood, *E* epidermis, *HFB* hypodermal fibre bundles, *HP* hypodermis, *LV* longitudinal vein, *MV* minor longitudinal vein, *S* stomata

Leaf anatomy changed slightly along the blade in relation to water-storage abilities i.e., the abundance of parenchymatic water-storage tissue (hydrenchyma). The basal, considerably thicker part of the leaf consists mainly of hydrenchyma traversed by a vascular system (Fig. 2a, b). It is protected by a layer of thick-walled epidermal cells covered with red resin, and underlain by a multi-layered hypodermis. As distance from the leaf base increases, the amount of water-storage tissue gradually decreases, the leaf surface is protected solely by the epidermis, covered with a cuticle, and the hypodermal fibre bundles are clearly visible (Fig. 2a,c,d).

**Fig. 3:**
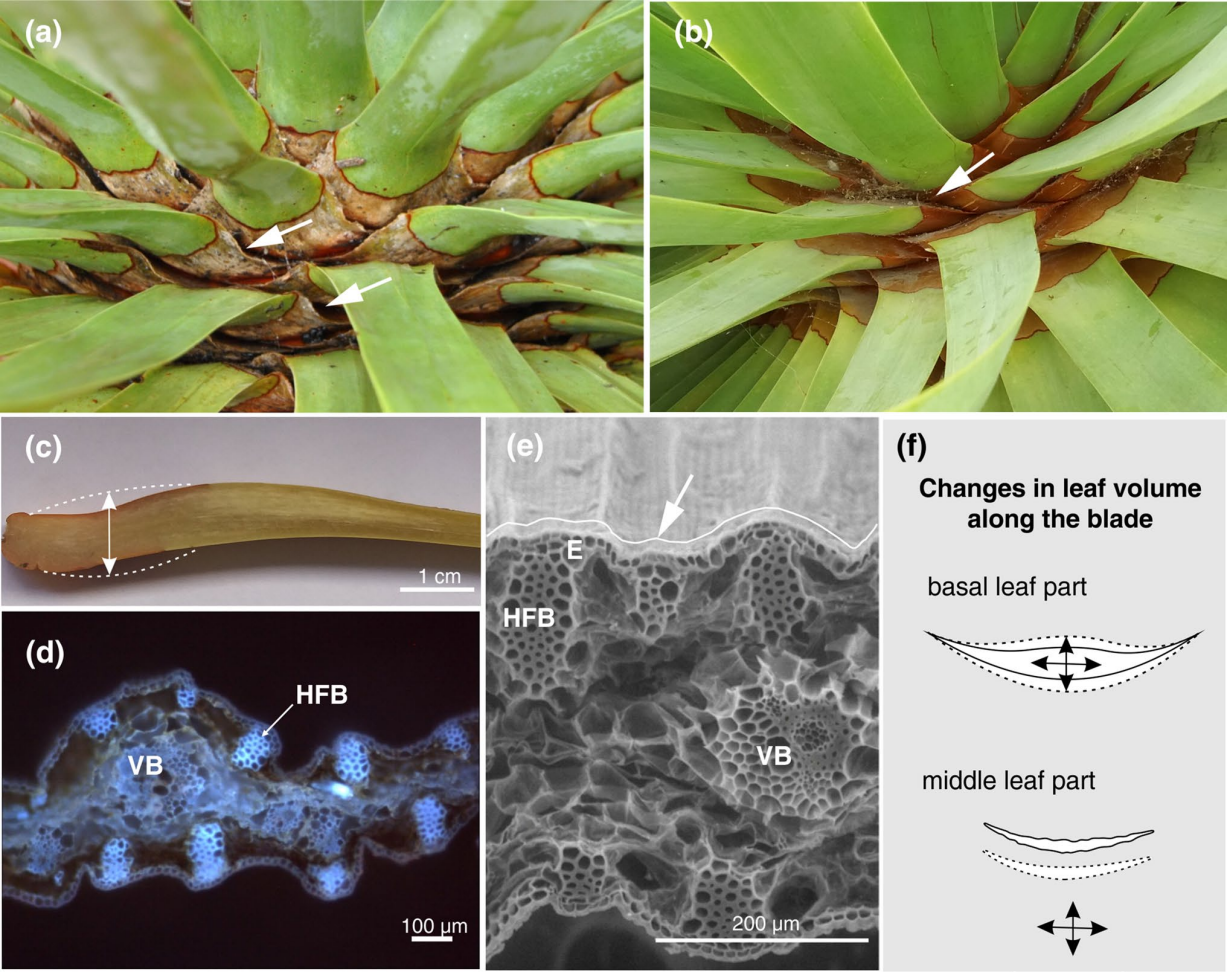
Changes in leaf morpho-anatomy with plant water content. **a** Thin, basal parts of leaves with low water content; free spaces between them are evident (arrows). **b** Thick, tightly overlapping leaf bases (arrow) in a well-hydrated plant. **c** Longitudinal, free-hand section of the basal part of a leaf with low water content showing the potential change in volume (marked by the dashed line). **d** Transverse section of the middle part of a dehydrated leaf observed under UV light; note the hypodermal fibre bundles (HFB), which function as a supporting skeleton. **e** SEM cross section of the middle part of a slightly dehydrated leaf, with the undulated surface marked with a white line and indicated by an arrow. **f** Schematic representation of the changes in the volume of the basal and middle parts of leaves in relation to leaf-water content (areas enclosed by solid lines are for dehydrated leaves and those within dashed lines are for leaves with high water content). *E* epidermis, *HFB* hypodermal fibre bundle, *VB* vascular bundle

Moreover, we observed that the morphology of *D. draco* leaves changes with leaf-water content. When the leaf-water content is low, the basal parts of leaves in the rosette are not tightly arranged and spaces between them are observed (Fig. 3a, c). A shortage of water also increased the visibility of superficial fibre bundles on the leaf surface, which form a skeleton supporting the living tissues. In comparison, when the leaf-water content is high, the basal parts of the leaves tightly overlap and the surfaces of the leaf blades appear smoother (Fig. 3b, f).

### Rosette water uptake

Analysing the patterns of water droplet adhesion to *D. draco* rosette leaves, we noticed that the contact of water on the leaf surface varied from a semicircular droplet on young, steeply angled leaves to patches of water on mature leaves (Fig. S2). To further understand the nature of water-leaf surface contact, we measured *θ*. Water droplet *θ* ranged from 0° to 130°, and was influenced by leaf age. The mature leaves were more wettable, both on the adaxial and abaxial leaf surfaces, than the young ones (Table 1).

**Table 1:** Mean, minimum and maximum (in brackets) values of the contact angle (*θ*) measurements for adaxial (AD) and abaxial (AB) leaf surfaces of young and mature leaves of *D. draco* rosettes

Leaves	*θ* (°)	
	AD	AB
Young	93.25 (11–130)	80.03 (13–117)
Mature	71.10 (0–119)	60.28 (0–105)

**Fig. 4:**
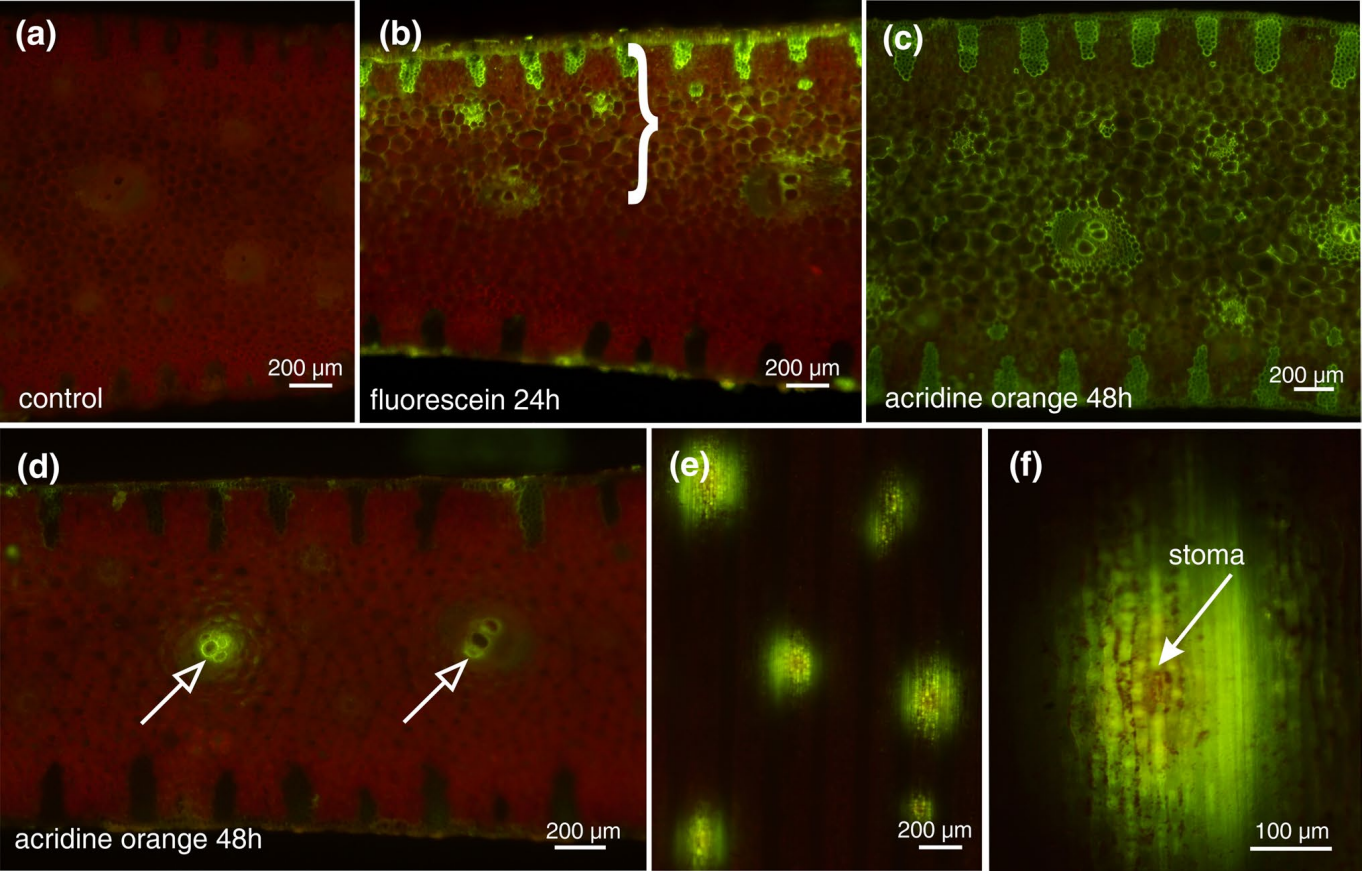
Evidence of water uptake in the middle region of a leaf visualised by fluorescent dyes. **a** Autofluorescence of a fresh, free-hand transverse section of the middle part of an untreated leaf. **b** Freehand transverse section of the middle part of a leaf, 24 h after exposure of the adaxial leaf surface to a solution of 0.01% fluorescein; fluorescent tracer distribution is marked by a bracket. **c** Freehand transverse section of the middle part of a leaf 48 h after exposure of both sides of the leaf to a solution of 0.01% acridine orange. Fluorescent tracer is visible throughout the entire section. **d** Transverse section of the same leaf at a distance of 5 mm from the place of dye application; dye is visible in vascular tissues only (arrows). **e**, **f** Adaxial surface of the leaf 48 h after acridine orange application; points of dye penetration include stomata, enlarged in (**f**)

We noticed also that water penetration into leaf tissues was possible on both the adaxial and abaxial sides of *D. draco* rosette leaves. Fluorescein applied to the adaxial surface at the mid-leaf diffused into the tissues, moving through apoplastic routes to the parenchymatic cells of the hydrenchyma (Fig. 4a). Similarly, acridine orange applied on both leaf surfaces entered the mesophyll, probably via the stomata (Fig. 4d, e) and, after entering the vascular system, was transported via xylem (Fig. 4c).

## Discussion

Fog may play a relevant ecological role as an alternative water source for plants (Burgess and Dawson 2004; Dawson and Goldsmith 2018; Schreel and Steppe 2020). *D. draco* rosettes possess the morphology of efficient fog harvester, as presented by Martorell and Ezcurra (2007). The numerous, (*ca*. 155) long leaves (*ca*. 70 cm in length) which are separated from each other at their tips, avoid fog-shadowing. Leaves are flexible (Marrero et al. 1998) and located high above the ground, thus they whisk in the wind, enabling better contact between the droplets in fog and leaf surfaces within the rosette. Nadezhdina and Nadezhdin (2017) reported the possibility of water uptake through leaf axils of young *D. draco*, assuming that leaf blades are rather hydrophobic. It seems, however, that restriction of water uptake to leaf axils of *D. draco* could be a disadvantage, especially for older, branched individuals, whose rosettes usually are not set upright. Moreover, the inclination of leaves within the rosette increases with leaf age. Thus, for some rosettes of *D. draco*, collecting and absorbing water at the leaf axils would be impossible. This hypothetical problem was resolved by the possibility of water uptake along the entire leaf of *D. draco*, as presented in our study.

For foliar absorption of water to occur, the leaf surface must be wettable, i.e., the contact angle between the water droplet and leaf surface should be less than 90^°^. The way in which water droplets adhere to leaf surfaces depends on the physical and chemical properties of the leaf, including the presence of trichomes and the nature of the leaf wax structure (Taylor 2011; Fernández et al. 2014, 2016). *D. draco* leaves are glabrous (Klimko and Wiland-Szymańska 2008), which means they are less water repellent than leaves with trichomes (Brewer et al. 1991). The *θ* of water on *D. draco* leaf surfaces decreased with leaf age, and this is in accordance with the observation of e.g. Neinhuis and Barthlott (1998) or Fernández et al. (2014) who measured *θ* on leaves of different deciduous trees. Leaves of evergreen *D. draco* are long-lived and persist within the rosette for more than a year, so during their life span, they are exposed to weathering by many environmental factors, such as heavy winter precipitation or high northeast trade winds, sometimes carrying sand from the Sahara desert. These agents may abrade the cuticle and increase *D. draco* leaf wettability, facilitating the direct uptake of water. The random and non-uniform action of the abrading factors seems to contribute to the heterogeneity of the leaf surface and great variation in *θ* values. Our data on leaf wettability of *D. draco* were supported with observations of water droplet behaviour on leaves of individuals growing in Spain, however, more measurements should be done on leaves of dragon trees growing in situ to establish how broadly our conclusions apply.

Uptake of water is possible not only via leaf axils of *D. draco*, as noted by Nadezhdina and Nadezhdin (2017), but also in the middle part of the leaf, as we proved using fluorescent dye penetration experiments. Moreover, based on measurements of *θ* on the different parts of *D. draco* leaves, it is reasonable to assume that areas favouring water absorption may be present along the entire leaf blade. In general, water penetration of the leaf surface with no trichomes is possible through the stomata (Burghardt et al. 2012), through the cuticle or through scales (reviewed by Fernández et al. 2017). Taking into account the distribution of fluorescent dye on *D. draco* leaf surfaces, we assume that water enters the middle part of the leaf through the stomata, but this needs experimental verification.

According to Martorell and Ezcurra (2007), rosettes of slender leaves in xerophytic species obtain water directly from the atmosphere, and succulent tissues in several organs may have developed to store this absorbed moisture. *D. draco* may store water not only in the stem (Jura-Morawiec et al. 2015; Nadezhdina and Nadezhdin 2017), but also in the massive, basal parts of leaves, as indicated in our study. Klimko et al. (2018) observed hydrenchyma in the middle parts of *D. draco* leaves. Our study shows that the basal parts of leaves are water reservoirs as much as 3–4 times thicker than that in the middle region. Based on the results of Nadezhdina and Nadezhdin (2017), we assume that water absorbed by the leaves of *D. draco* first rehydrates stem water reserves, after which it is stored in the basal parts of leaves. When water availability decreases, moisture stored in the hydrenchyma of these basal leaf regions may be the main reservoir for maintaining a favourable water status in the photosynthetic tissue of the leaf.

Storage succulence requires investment in mechanical adaptations (Males 2017). The leaf blade of *D. draco* is supported by a net of hypodermal fibre bundles, but its basal part, which is the main leaf-water reservoir, is additionally protected by a multi-layered hypodermis and thick-walled endodermis cells, covered with red resin (dragon's blood). This resin is a natural barrier that probably reduces leaf-water evaporation and protects leaves from damage by insects, while also sealing the leaf scar after leaf abscission (Jura-Morawiec and Tulik 2016).

In summary, our study revealed that the surfaces of leaves from the rosettes of *D. draco* are wettable. The mature leaves of the rosette are more wettable than the young ones. Water absorption is possible by both, the adaxial and abaxial surfaces. The water-storage tissue (hydrenchyma) is not uniformly distributed along the leaf of *D. draco*, but instead is more abundant in the basal part of leaf, where it forms a massive water reservoir. This water reservoir changes its volume depending on water content. Our findings support the hypothesis that the occurrence of leaf-wetting events, like fog, positively affects water balance in *D. draco* by direct uptake of water deposited on leaf surfaces.

### *Author contribution statement*

JJ-M Study conception and design; morpho-anatomical studies; field observation; writing of the manuscript. JM contact angle measurements; dye traces experiments and data analysis.

## Electronic supplementary material

Below is the link to the electronic supplementary material.

Fig. S1 Scheme of a rosette at the tip of a branch with exemplified positions of leaves (1-4) used in water droplet contact angle (θ) measurements. Leaves differ in age and angle of attachment with respect to the rosette tip, i.e., 1-2 are young, steeply angled, 3-4 are mature, horizontal or at an obtuse angle

Fig. S2 Water droplet adherence of leaves of *D. draco*. a Young leaves near the rosette tip; water droplets adhere to leaves despite almost vertical leaf orientation. b Mature leaf with nearly horizontal orientation showing patches of water on the wettable, adaxial surface
