# Supplementary material for: Wettability, water absorption and water storage in rosette leaves of the dragon tree (Dracaena draco L.)
Source: Planta. 2020 Jul 28;252(2):30. doi: 10.1007/s00425-020-03433-y (PMC7387376; doi:10.1007/s00425-020-03433-y)
Supplement: Supplementary file 1 — Fig. S1 Scheme of a rosette at the tip of a branch with exemplified positions of leaves (1-4) used in water droplet contact angle (θ) measurements. Leaves differ in age and angle of attachment with respect to the rosette tip, i.e., 1-2 are young, steeply angled, 3-4 are mature, horizontal or at an obtuse angle [file 425_2020_3433_MOESM1_ESM.pdf]

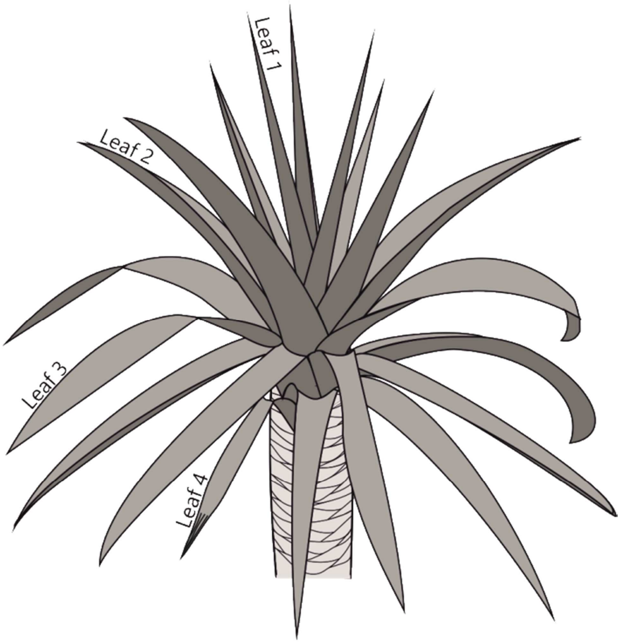

Fig. S1 Scheme of a rosette at the tip of a branch with exemplified positions of leaves (1-4) used in water droplet contact angle (θ) measurements. Leaves differ in age and angle of attachment with respect to the rosette tip, i.e., 1-2 are young, steeply angled, 3-4 are mature, horizontal or at an obtuse angle
