# Supplementary material for: Wettability, water absorption and water storage in rosette leaves of the dragon tree (Dracaena draco L.)
Source: Planta. 2020 Jul 28;252(2):30. doi: 10.1007/s00425-020-03433-y (PMC7387376; doi:10.1007/s00425-020-03433-y)
Supplement: Supplementary file 2 — Fig. S2 Water droplet adherence of leaves of D. draco. a Young leaves near the rosette tip; water droplets adhere to leaves despite almost vertical leaf orientation. b Mature leaf with nearly horizontal orientation showing patches of water on the wettable, adaxial surface [file 425_2020_3433_MOESM2_ESM.pdf]

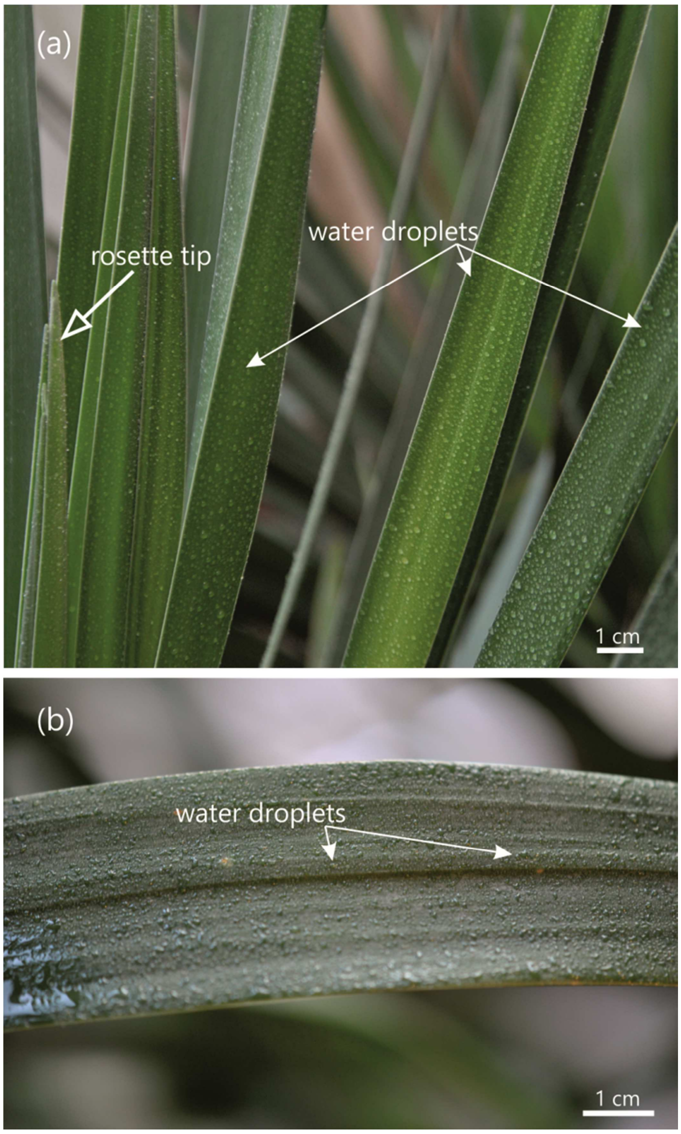

Fig. S2 Water droplet adherence of leaves of *D. draco*. **a** Young leaves near the rosette tip; water droplets adhere to leaves despite almost vertical leaf orientation. **b** Mature leaf with nearly horizontal orientation showing patches of water on the wettable, adaxial surface
